# Spurs in the Nose

**Published:** 1916-09

**Authors:** John C. Warbrick

**Affiliations:** Chicago


### Spurs in the Nose

By JOHN C. WARBRICK, M. D., Chicago.

Spurs in the nasal passages may be somewhat likened to the small outshoots growing on the limbs of an apple tree or to a peculiar fungous growth semi-circular in form often found attached to some object and again to the black knot growths commonly observed on unhealthy cherry trees.

Some of them may also be likened to the strong sharp spurs seen on the legs of most roosters, only a little smaller in size and of a different shape.

As is well known spurs are projections of a cartilaginous nature in many cases, while in other instances they seem to be formed of a combination of cartilage and bone, making a solid mass at times, almost as hard as stone and somewhat difficult to remove from its attachment.

Occasionally a small spur may be found of much softer consistence than cartilage, and also a larger one may be noted as well. These numerous outgrowths would seem to be very common in the nasal passages for some reason or other, while almost as a rule their origin is confined entirely to some part of the nasal septum, and they seldom if ever occur in any other part of the nostrils. Possibly they are more frequently noted along the base of the septum from the anterior part to the posterior extremity than any other place, while they may occur much oftener in the anterior half of the thin partition along the base than in the posterior half.

On the upper part of the nasal septum it would seem that spurs of any kind do not occur nearly so often as on the lower part. Just what the origin of these common protrusions is does not seem to be exactly known, although some theories may be put forth to explain them.

It may be they grow from some constant irritation, in a way not explained, or from congestion extending over a long period, a foci being formed at a certain place where accretion begins and extends by degrees.

The septum being formed mostly of cartilaginous material above with its base resting firmly on a bony structure, known as the union of the two surfaces or wings of the superior maxillary bones, it is hardly to be wondered at if such numerous outgrowths should be formed mostly of cartilaginous principles more so along the base where the two formations meet to form in some cases a combination spur consisting of cartilage and bone. All forms and sizes of these leeches may be found in the nasal passages, from the smaller speck about the size of a pinhead to the large and irregular one with shelving edge almost filling up the lower part of the cavity reaching backwards as it does and extending across the passage to project into the other side and become fixed.

These shelving outgrowths or elongated structures may begin at the base of the septum near the front part of the nose and extend to the posterior limits.

They may in some instances overlap the inferior turbinated body entirely, and almost covering it up so it cannot be seen, while in other cases they may in part transfix it.

In some cases an elongated spur may project into the middle turbinated body and to some extent transfix it.

A peculiar spur, something like a ladder, may occasionally be seen on the septum towards the upper part.

It begins at the front of the nose near the septal base and gradually extends in an upward direction projecting a little from the side and seeming to form numerous steps one above the other from its having a somewhat corrugated appearance. There is not very much evidence to prove that spurs are found any more frequently in one side of the nasal cavity than the other, although it might be the case, however, there does not seem to be any special reason why this should be so unless from some peculiar circumstances that may arise now and again.

They are, as has been often observed, found repeatedly growing in both nasal passages, time and time again it may be said almost without number, while they may be either single or multiple in number.

They may occur from almost any age in childhood up to that of an advanced period in life, and they may be noted in men, women and children of all ages.

Whether sex has any influence in determining the formation and growth of spurs, is not just known, nor whether they are found any more frequent in men than in women.

Possibly they occur in the nasal passages of one sex nearly as often as in the other, if the real facts were tabulated for comparison.

They may be found among people of all nationalities, and of various occupations, while it may be that very few are entirely free from one or more of them in their nostrils at some time during the course of their existence.

All shapes may be assumed by these septal barnacles. They may be oval, oblong, round, inclined, straight, corrugated, irregular, spear shaped, etc.

The edges of some of them may be sharp and pointed or sharp and shelving or blunt and thick.

It is hardly natural to have such objects in the nostrils, whether small or large in size, where they would seem to act like a foreign body, and in many instances tend to block them up and, at the same time obstruct the flow of mucous.

They are covered by mucous membrane all over and extend the extent of its surface in the nasal passages to some degree. While they seem to have a good supply of blood in most cases, for some of them when removed tend to bleed profusely, which may not be easy to check just at once.

Most spurs should be taken out of the nostrils, and more so if they are large in size and causing trouble, which, of course, they do. Some of them tend to bleed readily from a slight cause, such as dust getting in the passage or from increased tension of any kind in the blood vessels.

A small spur may cause little or no trouble of any kind in the nostril nor obstruct the breathing at all, but they tend to increase in size all the time, and by degrees reach a good proportion until some symptoms may be produced, then it is necessary to think of an operation.

A small spur is not difficult to remove, especially if it is situated in the front part of the nostril, and the only trouble that might be caused, is bleeding in some cases. A large spur may not be easy to remove, and more so if it is of a bony nature and of large surface and with a shelving edge. Some spurs of good size may be taken out of the nostrils without much bother, while others cause a great deal of difficulty, in some cases where patient cannot stand much pain or irritation, so that it has to be removed by degrees.

---

**Human Rabies in California.** J. C. Geiger, Calif. State Jour., June. The first case was reported in 1899. 33 others have occurred since 1909, all fatal. 9 had received Pasteur treatment but only 3 for what Pasteur considered the necessary length of time. The interval between the bite and death ranged from 16 days to 11 weeks. All cases were directly due to dog bites except one, due to a cat. For the year ending Mch. 31, 1914, (ibid. July) 428 brains of animals were received for examination in the State Laboratory, 9 being too much decomposed for examination. 70 of the remainder gave negative results, 339 showed Negri bodies and 9 more proved positive by inoculation experiments. The 348 demonstrated cases included 317 dogs, 14 cows, 11 cats, 4 horses, 1 goat and 1 coyote. These had bitten 235 human beings and 207 animals. 641 persons have received Pasteur treatment, only 3 being failures.

---

**Fecundity.** A French-Canadian gave birth to triplets after 8 months, April 14, 1914; to triplets after 6½ months, Dec. 31, 1914; to twins, full term, June 14, 1916. E. F. Fluhmann, Kenogami, Que., Med. World, Aug.

